# Trail Communication Regulated by Two Trail Pheromone Components in the Fungus-Growing Termite *Odontotermes formosanus* (Shiraki)

**DOI:** 10.1371/journal.pone.0090906

**Published:** 2014-03-26

**Authors:** Ping Wen, Bao-Zhong Ji, David Sillam-Dussès

**Affiliations:** 1 College of Forest Resources and Environment, Nanjing Forestry University, Nanjing, China; 2 IRD, UMR 211 BIOEMCO IBIOS, Bondy, France; 3 Université Paris 13, Sorbonne Paris Cité, LEEC, EA4443, Villetaneuse, France; 4 Jiangsu Agrochem Laboratory, Changzhou, China; 5 College of Agriculture and Biotechnology, Zhejiang University, Hangzhou, China; South China Agricultural University, China

## Abstract

The eusocial termites are well accomplished in chemical communication, but how they achieve the communication using trace amount of no more than two pheromone components is mostly unknown. In this study, the foraging process and trail pheromones of the fungus-growing termite *Odontotermes formosanus* (Shiraki) were systematically studied and monitored in real-time using a combination of techniques, including video analysis, solid-phase microextraction, gas chromatography coupled with either mass spectrometry or an electroantennographic detector, and bioassays. The trail pheromone components in foraging workers were (3*Z*)-dodec-3-en-1-ol and (3*Z*,6*Z*)-dodeca-3,6-dien-1-ol secreted by their sternal glands. Interestingly, ratio of the two components changed according to the behaviors that the termites were displaying. This situation only occurs in termites whereas ratios of pheromone components are fixed and species-specific for other insect cuticular glands. Moreover, in bioassays, the active thresholds of the two components ranged from 1 fg/cm to 10 pg/cm according to the behavioral contexts or the pheromonal exposure of tested workers. The two components did not act in synergy. (3*Z*)-Dodec-3-en-1-ol induced orientation behavior of termites that explore their environment, whereas (3*Z*,6*Z*)-dodeca-3,6-dien-1-ol had both an orientation effect and a recruitment effect when food was discovered. The trail pheromone of *O. formosanus* was regulated both quantitatively by the increasing number of workers involved in the early phases of foraging process, and qualitatively by the change in ratio of the two pheromone components on sternal glandular cuticle in the food-collecting workers. In bioassays, the responses of workers to the pheromone were also affected by the variation in pheromone concentration and component ratio in the microenvironment. Thus, this termite could exchange more information with nestmates using the traces of the two trail pheromone components that can be easily regulated within a limited microenvironment formed by the tunnels or chambers.

## Introduction

Pheromones are crucial for maintaining the cohesion of a termite colony. Information about trail direction or food quantity is given by trail pheromones [1] and sometimes by vibrations [2]–[4]. Differences between exploratory trails and recruitment trails, as well as the species-specific trail communication, have been observed many times in termites [5]–[8], despite the conservative chemical nature of the termite trail pheromones [9]. In bioassays, these differences could be both quantitative and/or qualitative [6], [7], [10]. In chemical analysis, it is still not clear if the exploratory trail and the recruitment (or pre-recruitment) trail are quantitatively and/or qualitatively different in chemical nature in many higher fungus-growing termites. In insects, the component ratio of most pheromones emitted by cuticular glands is fixed in individual species or subspecies to ensure isolation [11]–[13]. However, in social behavior, the communication among individuals needs to be precise, resulting in both qualitative and quantitative regulation of trail pheromones. In many recent studies, multi-component trail pheromones were found in several termite species, as reviewed in reference [9], but how they express as much information using few pheromone components in only trace amounts is not known.

In contrast to light-sensitive eusocial ants, termites rely on their trails to orientate since they are blind and they usually build tubes or shelters to cover their trails. Therefore, trail pheromones are vital for those termites (i.e., all Hodotermitidae, some Rhinotermitidae, Serritermitidae and most Termitidae) [14] that forage outside their nest to collect cellulose resources, with the exception of workers of *Hodotermes mossambicus*, which also use optical cues in addition to pheromone trails to orientate [15]. Although little is known of the foraging behaviors of cryptic termite species, open or semi-open field foraging in termites is common in many tropical species, such as some Macrotermitinae [16], Nasutitermitinae [17]–[21], and even in some basal termites, such as Hodotermitidae [22] and Rhinotermitidae [5], [23]. According to observations [5], [23], searching termites always travel slowly and attach their abdominal cuticle to the ground to lay exploratory trails. The increase in the number of termites on the foraging trail is obvious, once food has been encountered. Subterranean termites usually build straight tunnels underground [24], and they build mud shelters over trails when they are foraging in the open air. It is thought that the mud shelters are used to protect the foraging termites. The benefit from the microenvironments formed by mud shelters in chemical communication is rarely known while bioassays for recruitment pheromone always use termites regardless of their microenvironments such as pheromone exposure, moisture, etc.

Polyethism in the foraging process of termites is regulated by trail pheromones. Workers and soldiers are all capable of initiating the foraging behavior, but only workers or soldiers can recruit their nestmates [25], [26]. This caste-specific polyethism is primarily based on a difference in trail pheromone sensitivity between workers and soldiers and on a quantitative difference in pheromone production between castes [27], given that the sternal glands of soldiers are smaller than those of the workers in some species [26], [28]. However, further work is required on the caste-related qualitative differences in pheromone production. Moreover, the search for food is generally carried out by the oldest foragers [9], which have the most active sternal glands, but behavioral differences in workers of different nutritional levels remain to be analyzed.

The higher fungus-growing termite *Odontotermes formosanus* (Macrotermitinae) is an important Asian termite pest of seedlings, timbers and dykes. Trail communication in *O. formosanus* is highly effective in the network of tunnels built by workers. How this higher fungus-growing termite achieves the efficient trail communication using its trail pheromone is mysterious. Its sex-pairing pheromones have already been identified as (3*Z*)-dodec-3-en-1-ol and (3*Z*,6*Z*)-dodeca-3,6-dien-1-ol [29]; the trail pheromone of this species is known to contain an unsaturated alcohol secreted from the sternal gland located between the 4th and 5th sternites [30]; and the trail pheromone candidate (3*Z*,6*Z*)-dodeca-3,6-dien-1-ol has been shown to be as attractive as worker extracts [30], [31], but little is known of its communication using trail pheromones.

In this study, we investigated the chemical nature and the deposition and response regulation of the trail pheromone during the foraging of *O. formosanus* by using a combination of different techniques, including video analysis, solid-phase microextraction (SPME), gas chromatography (GC), gas chromatography coupled with mass spectrometry (GC-MS), GC coupled with electroantennographic detection (GC-EAD), and open-field trail-following bioassays.

## Results

### Exploration Behavior and Foraging Activity

**Figure 1:**
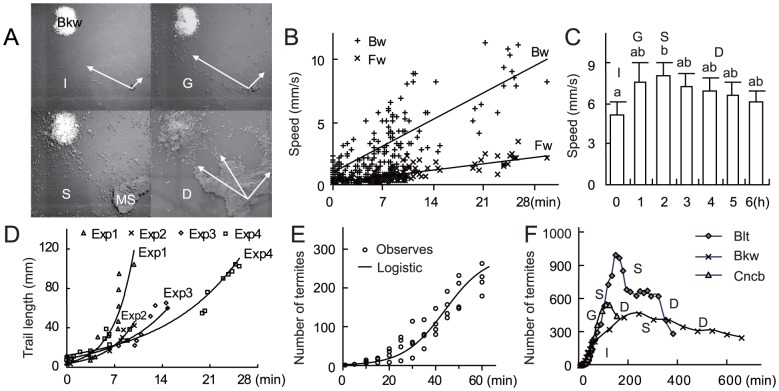
Dynamics of the walking speed, distance and number of workers in the searching and food-collecting process in *O. formosanus.* The error bar in each column indicates the standard deviation. (A) Typical performance of the four phases in the foraging process, using buckwheat powder (Bkw) as food. I phase with workers searching in fixed directions (arrows); G phase with the recruitment of workers after the food was discovered; S phase with workers going back and forth between the nest and the food under the protection of the mud shelters (MS); D phase with fewer workers collecting food under the mud shelters. (B) Speed dynamics of the searching workers in the first 30 min of each searching cycle. Number of searching termites tracked in the video file was 170 from 9 experiments. The walking speed of workers while searching forward (Fw) away from the nest increased linearly (*R^2^* = 0.61), and the speed of the workers returning to the nest (Bw) increases linearly (*R^2^* = 0.63). (C) Speed dynamics of the workers throughout the foraging process. *N* = 20 for each column; those columns marked with different small letters are significantly different (*P <*0.001). (D)Time-based increase of the trail length walked by each pioneer termite in the arena. In the analyzed 4 experiments (Exp 1 to 4), the trail lengths was extended by pioneer termites in exponential trends (From Exp 1 to 4, numbers of traced pioneer termites were 13, 8, 10, and 20, *R^2^* = 0.90, 0.97, 0.90, and 0.90, respectively). (E) The number of termites in the foraging arena in the I phase before the food was found (sampled every 5 min, from 10 experiments). If not disturbed, the number of searching termites in the arena increased logistically (*N* = 48, *R^2^* = 0.91) with an upper bound of 280. (F) Dynamics of the worker population before the mud shelters were built throughout the searching and food-collecting process in the presence of three types of food: Bkw, corncob (Cncb), or bark of the locust tree *Robinia pseudoacacia* (Blt). After the number of termites had increased logistically in the I phase, termites were recruited intensively with more worker encountered the food in G, S and D phase of foraging. The chaotic running was judged by the ratio of number of termites away from/on a trail. When the ratio was up to 30%, chaotic running was defined, and the phenomenon of chaotic running synchronized with surge of termite in S phase.

Behavioral analysis results of the recorded video files showed four successive phases in the foraging process with a 0.4 g food bulk: initial phase (I, 30–60 min), growing phase (G, 50–150 min), surging phase (S,100–200 min), and descending phase (D, > 200 min) (*n* = 10) (Figure 1A, C, and F). Soldiers and workers foraged at a ratio of 1: 7 to 1: 15 (soldier: worker, *n* = 15) in the search for food, and at a ratio of 1: 260 (soldier: worker, *n* = 10, P > 0.05) when collecting food. During the search for food, workers increased their speed with the time that they spent outside the nest. Workers that did not find food returned to the nest even if their search was short (0.1–2 min, *n* = 170). Their return speed was always higher than their outward speed and accelerated with the increasing amount of searching and trailing time since the first worker emerged from the nest (Figure 1B). Pioneer termites extended the trail length in an exponential way with time (Figure 1D). The number of individuals leaving the nest to follow the searching trail increased logistically (Figure 1E), before they reached the food. In the three phases after the food was discovered, the speed of workers increased slightly, but was only significantly higher in the S phase than in the G and D phases (*n* = 20, P<0.001) (Figure 1C). No difference was observed between the forward speed and the backward speed of workers (P = 0.65>0.05). However, during the D phase, workers contacting one another walked more slowly on the trail (4.64±0.14 mm/s, *n* = 145) than workers going back and forth on the trail (8.48±0.27 mm/s, *n* = 200, P<0.001). Also, the dynamics of the foraging population slightly changed in response to the types of food provided during the G, S, and D phases. More individuals fed on bark of the locust tree *Robinia pseudoacacia* than on corncob or buckwheat when an equal weight of these kinds of food was provided in the foraging arena (Figure 1F).

### Chemical and Electrophysiological Nature of the Trail Pheromone

**Figure 2:**
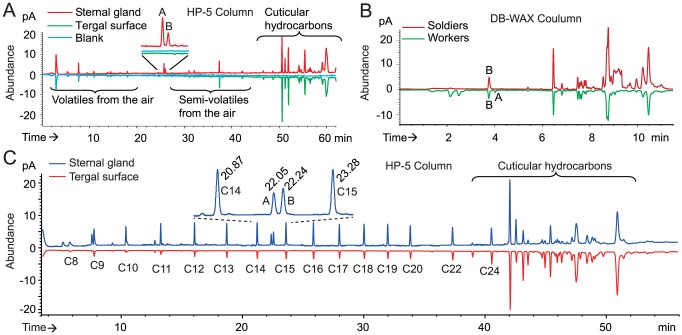
GC analysis of *O. formosanus* worker and soldier sternal gland extracts. (A) Comparative analysis of a 50 sternal gland extract (top) and the tergal surface (bottom) of 50 D phase workers on an HP-5 column, showing that peaks A and B are glandular specific. (B) Comparative analysis of a 15 searching worker gland extract and a 15 soldier gland extract on a DB-WAX column, showing that only compound B was present in soldiers, whereas both compounds A and B were present in workers. (C) Co-injection of *n*-alkanes to calculate the linear retention indices (LRIs) on an HP-5 column. The chromatogram was generated from an extract from a total of 60 trailing workers. LRIs of A and B were 1449 and 1457, respectively.

**Figure 3:**
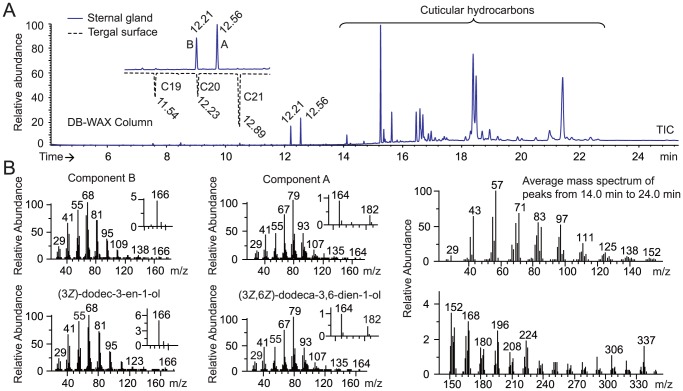
GC-MS analysis of a 135 *O. formosanus* worker sternal glands extract on a DB-WAX column. (A) Co-injection of *n*-alkanes to calculate the linear retention indices (LRIs) on a DB-WAX column. LRIs of A and B were 1996 and 2047, respectively. (B) Mass spectra of peaks A and B separated corresponded to those of (3*Z*)-dodec-3-en-1-ol and (3*Z*,6*Z*)-dodeca-3,6-dien-1-ol, respectively. Other peaks were contaminants from air or cuticular hydrocarbons according to their mass spectra.

**Figure 4:**
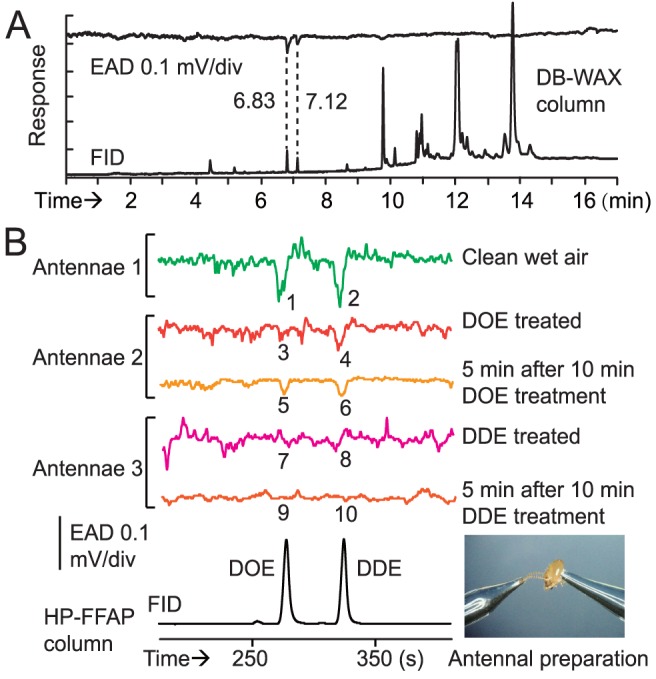
Antennal electrophysiological responses to the pheromone components. (A) The antennal response of *O. formosanus* workers to a 60 sternal gland extract on a DB-WAX column showing a significant and reproducible response of the antennae at the retention times 6.83 min and 7.12 min (Y-scale EAD  =  1.0 mV/div, Y-scale FID  =  2.0 mV/div), the same retention times for synthetic (3*Z*)-dodec-3-en-1-ol (DOE) and (3*Z*,6*Z*)-dodeca-3,6-dien-1-ol (DDE), respectively. (B) The antennae of workers of *O. formosanus* showing significant responses (peaks: 1, –0.08 mV; 2, –0.08 mV; 4, –0.06 mV, 5, –0.04 mV; and 6, –0.06 mV, T-test, P<0.001, n = 10) to DOE and/or to DDE when or 5 min after being exposed to DOE atmosphere (0.05 ng/mL for 10 min) or DDE atmosphere (same conditions) or not treated (the non-significant peaks being 3, 7, 8, 9 and 10). The column used was an HP-FFAP column.

After SPME-GC and SPME-GC-MS analysis, the comparison of the compounds presented on the surface of the sternal gland and on the tergal surface in workers and soldiers highlighted the presence of two compounds specific to the worker sternal gland and one compound specific to the soldier sternal gland (Figure 2A and B, Figure S1, and Table S2). The co-injection of the gland extract with a series of *n*-alkanes revealed that the linear retention indices (LRIs) of the putative active compounds were 1996 and 2047 on a DB-WAX column (Figure 3A). The injection of the standards under the same conditions indicated that peaks A (LRI 1996) and B (LRI 2047) were (3*Z*)-dodec-3-en-1-ol and (3*Z*,6*Z*)-dodeca-3,6-dien-1-ol, respectively. The same procedure on an HP-5 column showed that the LRIs of these two peaks corresponded to (3*Z*,6*Z*)-dodeca-3,6-dien-1-ol (LRI 1449) and to (3*Z*)-dodec-3-en-1-ol (LRI 1457) (Figure 2C and Table S1). The mass spectra of these compounds were identical to those of the synthetic standards (Figure 3B). GC-EAD analysis showed that these two compounds were EAD active in workers (Figure 4A). Experiments made with antennae maintained under (3*Z*)-dodec-3-en-1-ol exposure (0.05 ng/ml for 4–5 min) showed that the antennae were slightly sensitive to (3*Z*,6*Z*)-dodeca-3,6-dien-1-ol and restored the sensitivity to (3*Z*)-dodec-3-en-1-ol and (3*Z*,6*Z*)-dodeca-3,6-dien-1-ol following the removal of (3*Z*)-dodec-3-en-1-ol after 10 min exposure to pheromone. The same experiments with 0.05 ng/ml (3*Z*,6*Z*)-dodeca-3,6-dien-1-ol exposure showed that the antennae were sensitive to neither (3*Z*,6*Z*)-dodeca-3,6-dien-1-ol nor (3*Z*)-dodec-3-en-1-ol, even following the removal of 3(*Z*,6*Z*)-dodeca-3,6-dien-1-ol after 10 min exposure to pheromone (Figure 4B). Therefore, it was likely that (3*Z*,6*Z*)-dodeca-3,6-dien-1-ol was able to remove the antennal responses for both components.

### Contents and Ratios of the Pheromone Components

**Figure 5:**
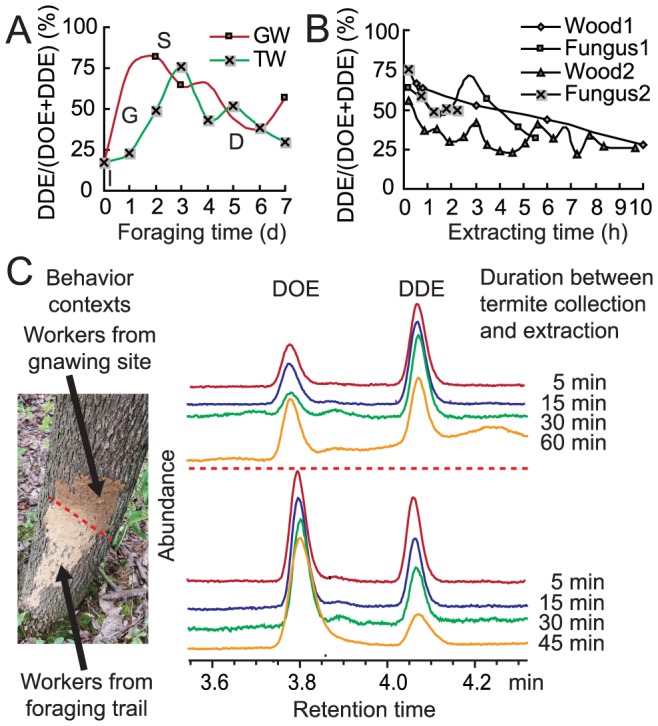
Fluctuations of pheromone content in workers of *O. formosanus* over time. DDE indicates (3*Z*,6*Z*)-dodeca-3,6-dien-1-ol; DOE indicates (3*Z*)-dodec-3-en-1-ol. (A) Monitoring experiment with in-site workers that were gnawing the food bulk (GW) or walking in the foraging trail (TW) during different phases of the foraging process (I, G, S, and D) in the experimental foraging arena when 8 g deadwood was provided. (B) DDE content in pheromonal secretion right after worker had fed on two types of food (wood, deadwood of *Liriodendron chinensis*; fungus, sporocarp of the wood decaying fungus *Gloeophyllum* spp). Experiments using termites collected from the same type of food were made twice (Wood 1 and 2, Fungus 1 and 2). DDE content was high during the gnawing (t = 0) and decreased after cessation of gnawing because of the collection of termites (t>0). (C) GC profile of the extraction from workers in and after exhibiting gnawing and trailing behavior. Ratio of the two pheromone components in the glandular extract changed steadily, but not too much within 5 min.

Ratios of the two pheromone components were quantified in foraging workers during different phases of their foraging activity (Figure 5A, B, and C). (3*Z*,6*Z*)-Dodeca-3,6-dien-1-ol increased during the G and S phases and slowly decreased or fluctuated during the D phase. Despite this fluctuation, quantifications of the extractions showed that the total amount of the two compounds in each sample was steady at approximately 1 ng per gland. Quantification was also carried out on workers under different behavioral contexts (Table 1 and Figure 5A, B, and C). Depending on their behavioral context, termites secreted different quantities of each pheromone component. Workers from sites that required more labour, such as workers in the core nest undertaking brood rearing, workers gnawing food, or building or repairing the mud shelters, secreted higher quantities of (3*Z*,6*Z*)-dodeca-3,6-dien-1-ol, whereas trail-laying workers secreted the most variable (3*Z*,6*Z*)-dodeca-3,6-dien-1-ol/(3*Z*)-dodec-3-en-1-ol ratios to regulate trail communication. When the termites were collecting food steadily during the D phase of foraging, they did not quantitatively regulate the pheromone in the foraging trail (Table 2).

**Table 1:** Component ratios of the trail pheromone in workers of *O. formosanus* under various behavior contexts.

Behavioral status and sites	Proportion of DDE:DOE	*Difference*	*N×M*
Gnawing in foraging arenas	1.58±0.52	a	13×13
Rearing larvae in core nests*	1.38±0.40	a	13×3
Trailing in foraging trails	1.01±0.77	ab	13×3
Building on food bulk	0.57±0.25	b	13×5
Searching on exploring trails	0.25±0.21	b	13×5
Fetching water in water arenas	0.24±0.21	b	13×3
Constructing new fungus gardens in satellite nests*	0.22±0.13	b	50×3
Gnawing workers from wild	1.72±0.52	a	13×4

DDE indicates (3*Z*,6*Z*)-dodeca-3,6-dien-1-ol; DOE indicates (3*Z*)-dodec-3-en-1-ol. Numbers of workers used in each extraction are indicated by *N*; numbers of replicates are indicated by *M*. Columns marked with same letters are not significantly different (*LSD* multiple comparison, *P*>0.05). Asterisk indicates data obtained from freshly collected nests. Number that follows the ± sign is a standard devia­tion.

**Table 2:** Quantification of the trail pheromone in the foraging trail of *O. formosanus* with trail-following bioassays in choice test with sternal gland extracts (SG) and (3*Z*)-dodec-3-en-1-ol (DOE).

Foraging time (h)	Pheromone (SG/cm)	DOE(ng/cm)
6	3 – 30	0.03 – 0.3
9	2 – 10	0.02 – 0.1
12	3 – 30	0.03 – 0.3
15	2 – 20	0.02 – 0.1

### Trail-following Behavior

Y-shape open-field trail-following bioassays with workers kept for 2 h in a Petri dish without food (defined as “activated”) showed that the active thresholds for (3*Z*)-dodec-3-en-1-ol and (3*Z*,6*Z*)-dodeca-3,6-dien-1-ol were both at 1 fg/cm (Table 3). A mixture of the two components at the threshold level did not induce a stronger trail-following activity. For the soldiers, the activity threshold for (3*Z*)-dodec-3-en-1-ol was also 1 fg/cm, whereas it was a little higher for (3*Z*,6*Z*)-dodeca-3,6-dien-1-ol (10 fg/cm) (Table 3). Trail-following bioassays using (3*Z*)-dodec-3-en-1-ol or (3*Z*,6*Z*)-dodeca-3,6-dien-1-ol at the base of the Y-shape showed that, when the concentration of the synthetic compound was between 1 and 10 fg/cm, workers preferred the trails made of (3*Z*)-dodec-3-en-1-ol. By contrast, when the concentration of the synthetic compound was 10^2^–10^4 ^fg/cm, the preference was for trails of (3*Z*,6*Z*)-dodeca-3,6-dien-1-ol. No synergistic effect was observed with the mixture of compounds. In addition, workers collected at the G, S, and D phases of foraging showed a preference for (3*Z*,6*Z*)-dodeca-3,6-dien-1-ol above 10^3^, 10^4^, and 10^2 ^fg/cm, respectively. Interestingly, activated workers were at least 10 times more sensitive to (3*Z*,6*Z*)-dodeca-3,6-dien-1-ol than workers directly collected during the D phase (Table 4). Activated workers preferred gland extracts from gnawing workers or (3*Z*,6*Z*)-dodeca-3,6-dien-1-ol than gland extracts from searching workers, (3*Z*)-dodec-3-en-1-ol or soldier gland extracts, when tested at the same concentrations (Table 5). However, workers of different nutritional levels showed no differences in behavioral reaction to the pheromones (Table 6). Finally, trail-following bioassays were performed with workers kept for 1 h in a Petri dish lined with filter paper impregnated with (3*Z*,6*Z*)-dodeca-3,6-dien-1-ol (Table 7). With a pre-treatment using 3.1×10^2^ to 3.1×10^3 ^pg/cm^2^ of the filter paper, the threshold concentration of (3*Z*,6*Z*)-dodeca-3,6-dien-1-ol was 10^4 ^fg/cm, which was identical to the threshold during the S phase of foraging. With a pre-treatment at 31.4 pg/cm^2^, the threshold was 10^3 ^fg/cm, which was the same as that during the G phase of foraging. Workers that could follow the (3*Z*,6*Z*)-dodeca-3,6-dien-1-ol base always chose the (3*Z*,6*Z*)-dodeca-3,6-dien-1-ol arm rather than the (3*Z*)-dodec-3-en-1-ol arm. Thus, habituation and depositing regulation in the trail pheromone of *O. formosanus* were observed.

**Table 3:** Y-shape trail-following bioassays using activated workers for active thresholds and synergistic effect.

Castes	Samples (fg, GEQ or WEQ/cm)		Active sample	Control	*n*	*Difference*
Worker	DOE	10^−2^	0 *ns*	0	15	–
		10^−1^	5 *ns*	0	15	b
		1	12**	0	15	–
		10^2^	15***	0	15	–
		10^4^	15***	0	15	–
	DDE	10^−2^	0 *ns*	0	15	b
		10^−1^	2 *ns*	0	15	b
		1	28***	0	30	–
		10	13**	0	15	–
		10^2^	15***	0	15	–
		10^3^	15***	0	15	–
		10^4^	15***	0	15	–
	DOE+DDE	10^−1^+10^−2^	0 *ns*	0	15	b
		10^−1^+10^−1^	10 *ns*	0	15	a
	GEQ	10^−5^	2 *ns*	0	15	–
		10^−4^	12**	0	15	–
		10^−3^	15***	0	15	–
		10^−2^	13**	0	15	–
		10^−1^	15***	0	15	–
	WEQ	10^−1^	0 *ns*	0	15	–
		0.5	3 *ns*	0	15	–
Soldier	DOE	10^−1^	3 *ns*	0	15	–
		1	15***	0	15	–
	DDE	1	8 *ns*	0	15	–
		10	14***	0	15	–

Samples were (3*Z*)-dodec-3-en-1-ol (DOE), (3*Z*,6*Z*)-dodeca-3,6-dien-1-ol (DDE), a blend of both compounds, a sternal gland extract (GEQ), an abdominal tergites extract (WEQ) in *O. formosanus* workers or soldiers. Hexane was used as control in the other branch. Selections were analyzed with a Mann-Whitney U-test with Continuity Correction (***P<0.001; **P<0.01; ns, not significant, *P* > 0.05). The columns marked with the same small letter were not significantly different (*Diff*) (*P* > 0.05); *n* indicated the total number of termites used.

**Table 4:** Open-field Y-shape trail-following bioassay with *O. formosanus* workers in different phases of foraging activity.

Phases	Branches (fg/cm)			Results			
	Base	DOE or DOE+DDE	DDE	≥Base	DOE or DOE+DDE	DDE	*n*
G	10 DDE	10	10	15 ns	14	1***	35
	10^2^ DDE	10^2^	10^2^	30	14	16	30
	10^3^ DDE	10^3^	10^3^	15	0	15***	16
S	10^3^ DDE	10^3^	10^3^	15 ns	7	8	50
	10^4^ DDE	10^4^	10^4^	15	1	14***	15
D	10 DDE	10	10	15 ns	6	9	28
	10^2^ DDE	10^2^	10^2^	16	2	14**	21
AD	1 DDE	1	1	15	15***	0	15
	1 DOE	1	1	15	15***	0	15
	10 DDE	10	10	18 ns	14**	4	30
	10 DOE	10	10	15	13**	2	15
	10^2^ DDE	20+80	10^2^	15	7	8	15
	10^2^ DDE	80+20	10^2^	16 ns	3	13**	26
	10^2^ DDE	10^2^	10^2^	15	1	14***	15
	10^2^ DOE	10^2^	10^2^	30	11	19	33
	10^3^ DOE	10^3^	10^3^	15	1	14***	16
	10^4^ DOE	10^4^	10^4^	30	7	23**	33

Four types of workers were used, G (growing phase), S (surging phase), D (descending phase), activated D phase workers (AD). Samples such as (3*Z*)-dodec-3-en-1-ol (DOE) or (3*Z*,6*Z*)-dodeca-3,6-dien-1-ol (DDE), mixture of standards (DOE+DDE in order) were laid down in the base and/or in one of the branch of the bioassay. The same protocol was used as in footnote of Table 3.

**Table 5:** Open-field Y-shape trail-following bioassays with *O. formosanus* activated D phase workers showing preference to recruitment pheromone.

Contrast (fg, GEQ/cm)		Results			
		≥Base	GWG	Contrast	*n*
DOE	10	15	15***	0	16
	10^2^	15	15***	0	17
	10^3^	15	12**	3	15
	10^4^	15	9 ns	6	15
DDE	10	15	15***	0	19
	10^2^	15	15***	0	16
	10^3^	15	5	10 ns	15
	10^4^	20	3	17***	20
SWG	0.001	15	15***	0	15
	0.01	30	25**	5	32
	0.1	17	3	14**	20
SDG	0.001	15	15***	0	16
	0.01	15	13**	2	18
	0.1	17	9 ns	8	20

The sample arrangements were 0.01 gnawing worker sternal gland extract (0.01 GWG/cm) laid in base and one arm, (3*Z*)-dodec-3-en-1-ol (DOE), (3*Z*,6*Z*)-dodeca-3,6-dien-1-ol (DDE), soldier sternal gland extract (SDG), and searching worker sternal gland extract (SWG) laid down in the other arm for contrast. The same protocol was used as in footnote of Table 3.

**Table 6:** Open-field Y-shape trail-following bioassays with workers of *O. formosanus* at different nutrition levels.

DOE or DDE (fg/cm)	Nutrition levels	≥Base	DOE	DDE	*n*
10	I	15 ns	6	9 *ns*	26
	II	15 ns	7	8 *ns*	35
	III	15 ns	6	9 *ns*	36
10^2^	I	15 ns	2	13**	21
	II	15 ns	1	14***	24
	III	16 ns	1	15***	28

The sample arrangements were (3*Z*)-dodec-3-en-1-ol (DOE) in one branch and (3*Z*,6*Z*)-dodeca-3,6-dien-1-ol (DDE) in the base and in the other branch at the same concentration. Worker from I to III, the former was less fed than the latter. The same protocol was used as in footnote of Table 3. Arm selection was calculated in ≥Base workers (that follow trails longer than the base branch). Significance of DDE arm selection was the statistical comparison between the total number of workers selected DDE arm and the number of workers going further than the base.

**Table 7:** Open-field Y-shape trail-following bioassays with (3*Z*,6*Z*)-dodeca-3,6-dien-1-ol exposed *O. formosanus* workers.

Trails (fg/cm)	Treatments (pg/cm^2^)	Results			
		≥DDE base	DOE	DDE	*n*
10^2^	31.4	16ns	2	14***	36
10^3^	31.4	20***	1	19***	20
	3.14×10^2^	18ns	3	15**	30
	3.14×10^3^	17ns	2	15**	39
10^4^	3.14×10^2^	15***	0	15***	16
	3.14×10^3^	16***	1	15***	16
10^5^	3.14×10^3^	15***	0	15***	15

(3*Z*)-Dodec-3-en-1-ol (DOE) or (3*Z*,6*Z*)-dodeca-3,6-dien-1-ol (DDE) can be applied in the base and/or in one of the branches at the same concentration. The same protocol was used as in footnote of Table 3. ≥ DDE base indicated the number of termites following a trail longer than the base and making a choice between the two branches. Significance of DDE base was the statistical comparison between the number of workers going further than the base and the total number of workers tested. Significance of arm was the statistical comparison between the number of termites choosing one or the other branch.

## Discussion

### Regulation of Pheromone Deposition

The nature of a termite recruitment trail pheromone has been the subject of much research. In this study, we identified the trail pheromone in *O. formosanus* workers to be a mixture of (3*Z*)-dodec-3-en-1-ol and (3*Z*,6*Z*)-dodeca-3,6-dien-1-ol, with behavior-related regulation of the ratio of the two components. Both compounds were secreted during all four phases of the foraging process. In the trail-following bioassays, the active thresholds for both compounds ranged from 1 fg/cm to 10 pg/cm, according to the behavioral contexts and pheromonal exposure of the termites investigated. No synergistic effect was observed between the two compounds. Our results suggest that (3*Z*)-dodec-3-en-1-ol orientates termites to explore their environment, whereas (3*Z*,6*Z*)-dodeca-3,6-dien-1-ol can both orientate and recruit termites to the food. This difference might be linked to the additional double bond of (3*Z*,6*Z*)-dodeca-3,6-dien-1-ol, which could provide this compound with a stronger affinity to bind to the pheromone receptors compared with the lone double bond of (3*Z*)-dodec-3-en-1-ol, based on the results of the GC-EAD experiments. The recruitment effect generated qualitatively by an ephemeral component alongside a persistent component has already been inferred in *Nasutitermes corniger* [8], [26]. Quantitative modulation of the recruitment effect by the concentration of the pheromone has been observed in *Reticulitermes* spp. [7], [32], [33], *Prorhinotermes* spp. [34], and *N. corniger* [8], [26]. In our analyses, both qualitative and quantitative pheromone modulations were identified in *O. formosanus*.

### Regulation in Behavioral Response to Pheromone

As the major component of the secretion of searching termites with lower active thresholds (Table 3), (3*Z*)-dodec-3-en-1-ol might act at a low concentration during the initial searching phase, when the termites explore their environment, whereas the minor (3*Z*,6*Z*)-dodeca-3,6-dien-1-ol does not induce a specific response. Later, the succession of termites walking on the exploring trails produces reinforcement of the pheromone concentration on these trails. After encountering food, together with the detection of phago-stimulating pheromones, the habituation of the termites to pheromone, observed in our bioassays, might be responsible for the retention of gnawing termites [35]. As the major component in pheromones produced by the first gnawing workers at the food source, (3*Z*,6*Z*)-dodeca-3,6-dien-1-ol acted at high concentrations during the food-collecting process and induced the chaotic distribution of termites during the surging phase. Workers followed trails comprising both compounds from the nest to the food with increasing speed until the number of gnawers exceeds a certain threshold when termites were led to run chaotically (Fig. 1A, F). It has already been demonstrated in several species that trail activity increases as a function of the number of termites laying the trail [7], [26]–[28], [33]. This mechanism prevents over-recruitment of termites to small gnawing sites. The chaotic distribution of surging *O. formosanus* foragers did not disturb the original trail made by the searching workers, but did help to smooth the foraging trail. Later, when the food was less abundant, the pheromonal secretion and response system was regulated to alleviate the recruitment effect, thus reducing the number of workers on the trail. Mud shelters were constructed by termites during the food-collecting phases, which suggested that the construction was intended mainly to protect the recruitments trails rather than to avoid predators. Mud shelters might be used to avoid diffusion of the pheromone and to control precisely the pheromone regulation. Based on a combination of our analyses and observations, it is possible to illustrate the regulation of (3*Z*)-dodec-3-en-1-ol and (3*Z*,6*Z*)-dodeca-3,6-dien-1-ol in the trail and arena by workers, based on the secreted componential content and the number of in-site termites (Figure S2).

### Prospects for the Research of Termite Pheromones

Our study clearly showed changes in the pheromone blend from the cuticular glands of *O. formosanus* depending on the behavioural contexts, which is the first report of this phenomenon for termites and for insects in general. Thus, the ratio of the components identified as trail pheromones in termites in previous studies [9] might have been misjudged. Some minor compounds might be detected only from workers within a specific behavioral context.

Moreover, the secretion of the compounds is regulated precisely by the termites, which could influence the species-specificity of sympatric termite species. For example, trail pheromones of the sympatric species *N. guayanae* and *N. voeltzkowi* comprise (3*Z*,6*Z*,8*E*)-dodeca-3,6,8-trien-1-ol and neocembrene. Their proportions vary according to the species [36] and may be regulated during the foraging process to enable species specificity of trails. Another strategy of species specificity is the use of additional compounds, as seen between *O. formosanus* and *Macrotermes barneyi*, the latter using only (3*Z*)-dodec-3-en-1-ol as a trail pheromone [37]. The chemical structure of the trail pheromone is the same than the one of the sex-pairing pheromone secreted by females of *O. formosanus* [29]. The identical chemical nature of the trail pheromone and the sex pheromone within a species has been reported in other species, such as (3*Z*,6*Z*,8*E*)-dodeca-3,6,8-trien-1-ol in *Psammotermes hybostoma* [38], and *syn*-4,6-dimethyldodecanal in *Zootermopsis nevadensis* and *Z. angusticollis* [39]. This parsimonious use of the pheromone compounds within termite species is reinforced by the use of the same compounds among different species. In the Macrotermitinae, the common major trail pheromone is (3*Z*)-dodec-3-en-1-ol [37], sometimes associated with (3*Z*,6*Z*)-dodeca-3,6-dien-1-ol or (3*Z*,6*Z*,8*E*)-dodeca-3,6,8-trien-1-ol as minor components [9]. It might be that higher termites have evolved an effective control of the pheromone biosynthesis pathways to regulate the component ratio in the cuticular sternal gland. The basal eusocial termites, which use trail pheromone components from a cuticular gland, show convergent evolution in the trail pheromone regulation in comparison with higher eusocial ants, which use different trail pheromone components from glandular reservoirs or exocrine glands [40].

For practical purpose, following the identification of the trail pheromones in this study and the sex-pairing pheromone of *O. formosanus* [29], it might be possible to develop a biological control system that uses the recruitment pheromone in the field to control this pest species through disruption of its foraging and other social behavior, as the overdose of recruitment pheromone leads to the loss of trail-following ability of foraging termites.

## Materials and Methods

Termite colonies were collected and used to build the experimental system for observation of the foraging behavior. Secreted pheromones in the artificial trail tunnel were extracted and analyzed. Pheromones from the glandular cuticle was collected with SPME method and analyzed by GC, GC-MS and GC-EAD for both quantitative and qualitative pheromone regulation. Sufficient pheromone from the glandular surface was gathered within 3 min to monitor the changes of pheromonal secretion under different behavioral contexts. Conditions such as caste, nutrient level, behavioral context, atmospheric pheromone, moist content and temperature, were considered and/or simulated in GC-EAD experiments and trail-following bioassays.

### Ethics Statement

With the permissions from the Administrative Office of School Industry in Nanjing Forestry University, the Administrative Office of Dr. Sun Yet-Sen’s Mausoleum, and the Nanjing Xinguiyuan Termite Pest Control Company, *O. formosanus* colonies from the Beidashan arboretum at Nanjing Forestry University (N32°04′50.71″, E118°49′19.16″), the dyke of Daxintang Reservoir in Yanxi Town, Gaochun County, Nanjing (N31°22′36.78″, E119°06′16.96″), and the dam of Qilian Reservoir in Qiaolin Town, Pukou District, Nanjing (N31°54′48.72″, E118°28′5.92″) were collected and used to build the experimental nest systems.

### Insects and Experimental System

*O. formosanus* builds a large nest comprising large cavities and small chambers linked by a network of galleries. The foraging distance of a colony has been recorded as covering 4 to 35 m [41], [42]. The termites build a circular gallery around the food to facilitate the activity of foragers [43]. In the field, there are always several foragers searching out of the terminal of the mud shelters tubes during the night. [44] (personal observation). Colonies containing the queen cell, the core fungus combs, and several satellite fungus combs were collected by digging each nest out rapidly and held in polypropylene containers (30 cm×50 cm×65 cm). Water, supplied by porous bricks, and food in half-sealed arenas were connected to the base of the nest container with glass tubes (1–2 cm in diameter) (Figure S3). The container was placed on sponge cushions and kept indoors at 22±3°C. A foraging arena (20 cm×20 cm×5cm) with a glass cover on top for observation was constructed with a tunnel connected to the nest. Once the colonies had settled following their transfer to the laboratory (the termites were seen to feed, fetch water and defecate steadily), a total of 5 colonies were used for behavior observation and pheromone extraction.

Workers used for bioassays were selected based on their activity in the foraging arena. Thus, we distinguished four foraging phases (Figure 1A, C, and F). The worker: soldier ratio was calculated based on the observations of the foraging termites. Food bulk (0.4 g) was provided in the semi-sealed foraging arena. The open-field foraging process was recorded under infrared lights using an infrared camera (Figure S3). The walking speed and number of individuals were determined by tracking the termites in the video file. The glass components of the arena were cleaned after each observation (10 times ultrasonic washing with pure water, and then heated to 210°C for 30 min).

### Solvent Extraction of Pheromone

Artificial trails (11 mm in diameter, 60 or 75 cm in length) with damp [Moisture Content (MC) 150–170%] and clean silica gel as substrate were set between the nest and the food arena, and a jelly mixture of agar and hay was used as food (Figure S3). Once the termites had discovered the food and had foraged steadily in the D phase for 6, 9, 12, and 15 h, the silica gel substrate was completely removed with 15–40 mL pure water and then successively extracted with hexane till the water showed no trail-following activity. The extracts were concentrated under a nitrogen flow and used for bioassays on dry filter paper (MC 14–17%).

In total, 50 worker sternal glands were dissected with iris scissors and extracted with 500 μL hexane for 1 h for comparison in the bioassays. Abdominal tergites were also dissected and extracted in the same way for comparative bioassays. Moreover, standards of (3*Z*)-dodec-3-en-1-ol dissolved in hexane in a concentration series were used in bioassays as an index for quantification.

### Chemical Analysis of the Pheromone

A termite was held in a clean air flow with forceps gripping the thorax to expose its sternites under a stereomicroscope. The terminal 1.0 mm part of a 65 μm PDMS/DVB fibre (Supelco, USA) was exposed and used to rub the glandular surface between the fourth and fifth sternites. The fibre was then desorbed in the injection port of the GC or GC-MS. The same method was used to rub the inter-tergal space of the abdomen of workers and soldiers as a control. An activated fibre was held under the air flow as a blank control. To monitor the secretion dynamics, the SPME fibre was used to rub the gland surface for 0.2 min per worker, and a total of 13–15 workers were extracted rapidly within 3 min after collection from their original site where they were exhibiting certain behaviors. The sternal gland contains only a single reservoir in this species [30]; therefore, the component ratio is unlikely to change substantially within 3 min (Figure 5C). The ratio of components in the extraction was used to represent the ratio of pheromone components on the gland surface.

GC and GC-MS analyses followed the conditions as described by Wen et al. [29] with modifications for fast GC quantifications to monitor the secretion in termites under various behavioral contexts. A DB-WAX column was used with 3 mL/min N_2_ as carrier gas. The oven ramp was 15°C/min from 135°C to 200°C. After two successive analyses, the oven was heated to 250°C and held for 5 min to clear any residues in the column. A series of 0.1, 0.5, 1.0 and 2.0 ng of (3*Z*)-dodec-3-en-1-ol and (3*Z*,6*Z*)-dodeca-3,6-dien-1-ol were injected for quantification. An HP7890N-5975A GC/MSD (Agilent, US) system was used. Data were analyzed using AMDIS software (NIST, US).

### Chemical Analysis of the Pheromone by GC-EAD

For GC-EAD analysis of the compounds, a quarter of the head with an antenna was cut from a living worker under a stereomicroscope with a razor blade. The terminal flagellum of the antenna was cut open vertically. Instrumental conditions were the same as described for alates of *O. formosanus* [29].

Another GC-EAD experiment was conducted to show antennal electrophysiological insensitivity in pheromone exposure. Antennal preparation is illustrated in Figure 5B. Ag/AgCl electrodes immersed in saline solution [29] in glass pipettes were used. A head capsule of a live worker was cut and connected to the tip of the pipette of the grounding electrode. The terminal flagella of the two antennae were connected to the tip of the pipette of the recording electrode. An HP-FFAP column (30 m×0.25 mm, 0.25 μm, Agilent J&W) was used with 3 mL/min N_2_ as carrier gas on an HP6890N GC (Agilent, US). The GC oven ramp was set as 150°C (2 min), then 8°C/min to 230°C. Immediately before injection of 100 ng of (3*Z*)-dodec-3-en-1-ol and 100 ng of (3*Z*,6*Z*)-dodeca-3,6-dien-1-ol SPME samples, a (3*Z*)-dodec-3-en-1-ol or (3*Z*,6*Z*)-dodeca-3,6-dien-1-ol dispenser at 2.5 ng/s (25°C) was mounted in the entrance of the Pasteur pipette (12 mm in diameter). Thus the antennae were exposed to a 0.05 ng/mL pheromonal atmosphere in the 44 cm/s clean wet air (RH 90%) flow before the separated GC elution reached the EAD system at 4.9 min [(3*Z*)-dodec-3-en-1-ol] and 5.6 min [(3*Z*,6*Z*)-dodeca-3,6-dien-1-ol]. Four fifth of the GC elution was conducted to the EAD using an OSS-2 column splitter (SGE, AU). Data were recorded using an IDAC4 (Syntech, NL) data collector and analyzed using GCEAD2011 software (Syntech, NL). After 10.0 min pheromone exposure, the dispenser was removed to enable the revival of antennae for another analysis within 30 min after the initial antennal preparation.

### Chemical Standards

(3*Z*)-Dodec-3-en-1-ol and (3*Z*,6*Z*)-dodeca-3,6-dien-1-ol were synthesized by Shanghai Youde Chemical Technology Co., LTD (Shanghai, China). Structures were confirmed by NMR spectrometry (Figure S4). The chemical shifts for synthetic (3*Z*)-dodec-3-en-1-ol were ^1^H NMR (400 MHz, CDCl_3_) δ 5.56, 5.36, 3.64, 2.33, 2.06, 1.52, 1.34, 1.30, 1.27, 0.88. The chemical shifts for (3*Z*,6*Z*)-dodeca-3,6-dien-1-ol were ^1^H NMR (400 MHz, CDCl_3_) δ 5.56, 5.52, 5.40, 5.35, 3.66, 2.82, 2.36, 2.05, 1.43, 1.38, 1.32, 1.27, 0.89.

### Trail-following Bioassays

Open-field Y-shaped trail-following bioassays were performed on damp filter paper discs with a 120° angle between each branch, as described by Sillam-Dussès et al. [38], Hall et al. [45], and Traniello et al. [8] (Figure S5). On the stem (3 cm) and/or on one of the Y branches (5 cm), a trail was drawn with a microliter syringe containing extract, standards or hexane control. Then, the filter paper was dampened to MC 170% with atomized pure water. One termite was deposited in a glass waiting chamber, the 3.5 mm wide opening being located at the base branch. The termites used for the bioassays were chosen based on conditions such as their foraging behavior contexts [phase I, G, S, D or activated D (AD, D phase workers held for two hours under clean and wet air flow without food after collection in open field)]; their nutrition level according to the amount and darkness of the food in their guts (termites at the level I, II, or III, the latter being more filled and darker from the former); or their atmospheric (3*Z*,6*Z*)-dodeca-3,6-dien-1-ol exposure in the Petri dish where termites were kept in for 1h (2, 20, or 200 ng of (3*Z*,6*Z*)-dodeca-3,6-dien-1-ol corresponding to 31.4, 3.14×10^2^, 3.14×10^3 ^pg/cm^2^ in the dish. Samples were impregnated on the wet filter paper in the Petri dish and evaporated to remove the solvent before the introduction of the termites). For behavior and caste-related bioassays (Table 5), sternal glands of workers from the food bulk, the searching trail, as well as soldiers from the water arena were dissected and extracted with hexane within 10 min after collection. Trails, followed by termites, longer than 3.0 cm were considered to be active, whereas those shorter than 3.0 cm were considered to be inactive. The filter paper and the tested termite were used only once, and the waiting chambers were cleaned by soaking them in pure water.

### Statistical Analyses

Measurements were analyzed and compared using Student’s t-test. Ratio of workers and soldiers were analyzed using Chi-square test. Selection was analyzed using Mann-Whitney U-test. Significance levels are indicated as follows: *P<0.05; **P<0.01; ***P<0.001; ns: not significant. *N*  =  number of tests if not otherwise mentioned.

## Supporting Information

Figure S1 **Comparative GC analysis of the glandular extract and tergal cuticle extract in the soldier caste of** ***O. formosanus*** **on a DB-WAX column.** One component was found to be specific to the glandular cuticular surface of the soldiers.
(TIF)

Figure S2 **Illustration of trail communication in the foraging behavior of** ***O. formosanus*** **.** I, G, S, and D indicate the four phases in the foraging behavior. ST is the searching trail. FT is the foraging trail. Workers went from the satellite nest (SN) to the foraging arena (FA) by following a foraging trail made of secreted (3*Z*)-dodec-3-en-1-ol (DOE) and (3*Z*,6*Z*)-dodeca-3,6-dien-1-ol (DDE). When the food was collected, workers went from the foraging arena to the satellite nest to feed termites in the nest or to construct fungus garden. The Y axis indicates the content of each pheromone component and the number of termites in the satellite nests, trails and arenas. Values are for illustration only.
(TIF)

Figure S3 **System for observation of the foraging behavior in an indoor** ***O. formosanus*** **nest.**
(TIF)

Figure S4 **^1^HNMR spectra of synthetic standards.**
(TIF)

Figure S5 **Trail-following bioassays.** A Y-shape trail-following bioassay apparatus comprising a piece of filter paper and a glass waiting chamber where a termite (T) was deposited. Trails were drawn with microsyringes containing sternal gland extract, (3*Z*)-dodec-3-en-1-ol (DOE), (3*Z*,6*Z*)-dodeca-3,6-dien-1-ol (DDE), or hexane control according to the testing purpose (active threshold, synergy test or selection test).
(TIF)

Table S1 **LRI calculation of the two sternal glandular specific components in** ***O. formosanus*** **.** Since both components were polarized, retention times or LRIs would change a lot (SE<10/0.5 year) with the bleeding of stationary phase on a DB-WAX capillary column.
(DOC)

Table S2 **Volatile contaminants identified in the GC-MS analysis.**
(DOC)
