# Supplementary figures and images for: Trail Communication Regulated by Two Trail Pheromone Components in the Fungus-Growing Termite Odontotermes formosanus (Shiraki)
Source: PLoS One. 2014 Mar 26;9(3):e90906. doi: 10.1371/journal.pone.0090906 (PMC3966735; doi:10.1371/journal.pone.0090906)

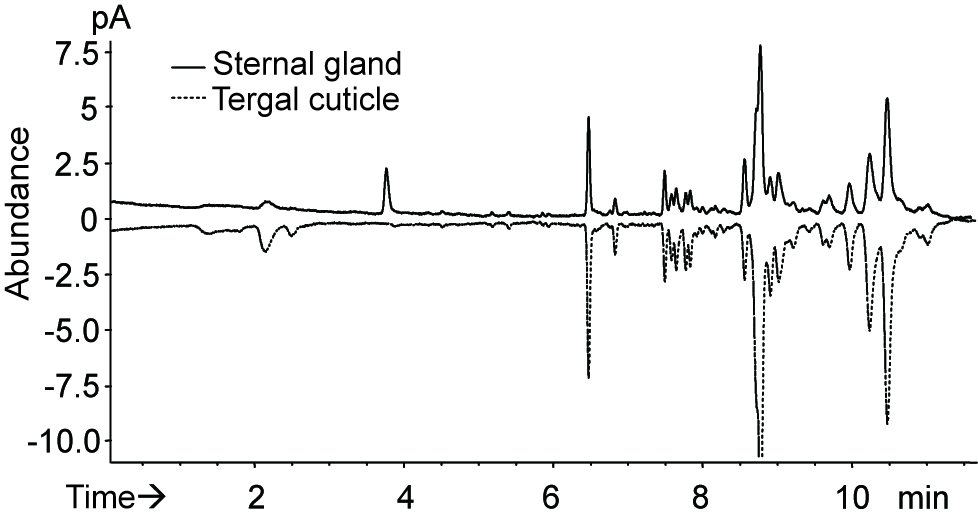

Supplement: Figure S1 — Comparative GC analysis of the glandular extract and tergal cuticle extract in the soldier caste of O. formosanus on a DB-WAX column. One component was found to be specific to the glandular cuticular surface of the soldiers. (TIF) [file pone.0090906.s001.tif]

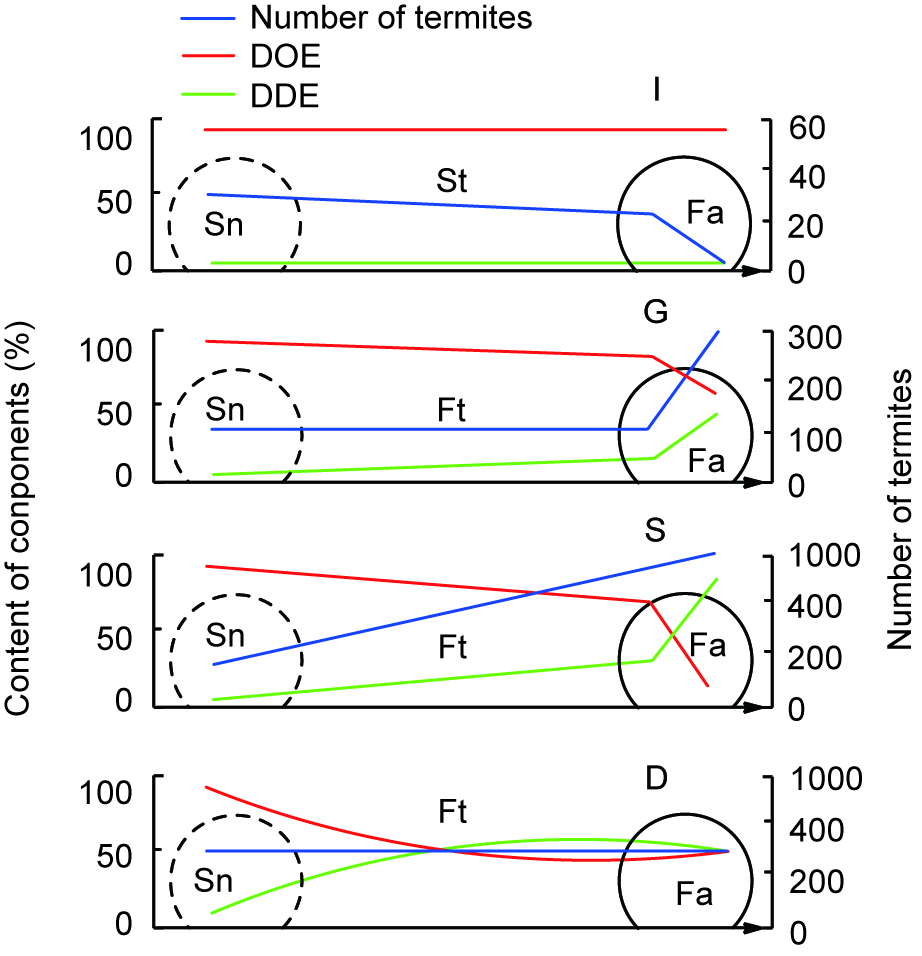

Supplement: Figure S2 — Illustration of trail communication in the foraging behavior of O. formosanus . I, G, S, and D indicate the four phases in the foraging behavior. ST is the searching trail. FT is the foraging trail. Workers went from the satellite nest (SN) to the foraging arena (FA) by following a foraging trail made of secreted (3Z)-dodec-3-en-1-ol (DOE) and (3Z,6Z)-dodeca-3,6-dien-1-ol (DDE). When the food was collected, workers went from the foraging arena to the satellite nest to feed termites in the nest or to construct fungus garden. The Y axis indicates the content of each pheromone component and the number of termites in the satellite nests, trails and arenas. Values are for illustration only. (TIF) [file pone.0090906.s002.tif]

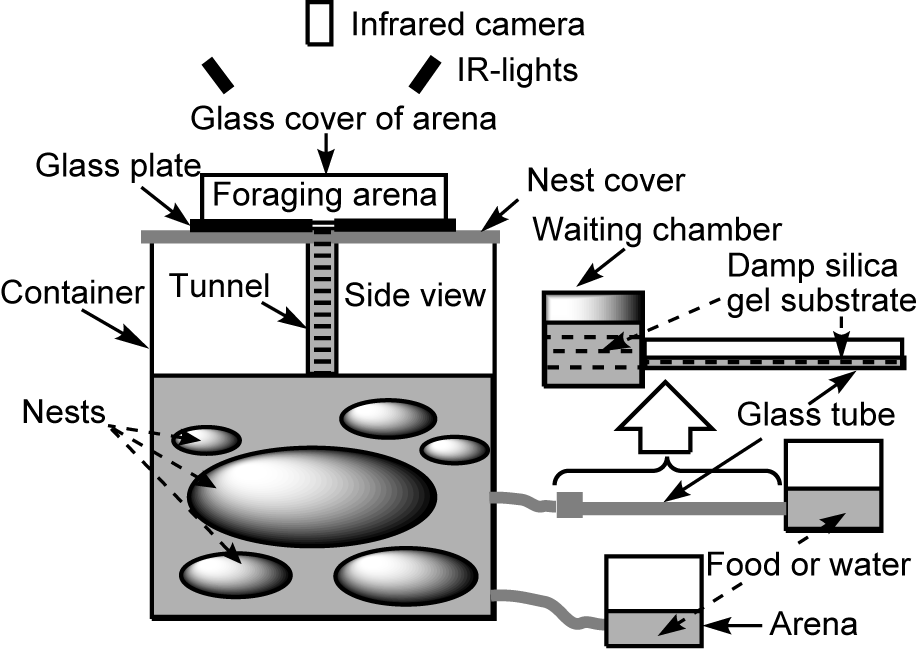

Supplement: Figure S3 — System for observation of the foraging behavior in an indoor O. formosanus nest. (TIF) [file pone.0090906.s003.tif]

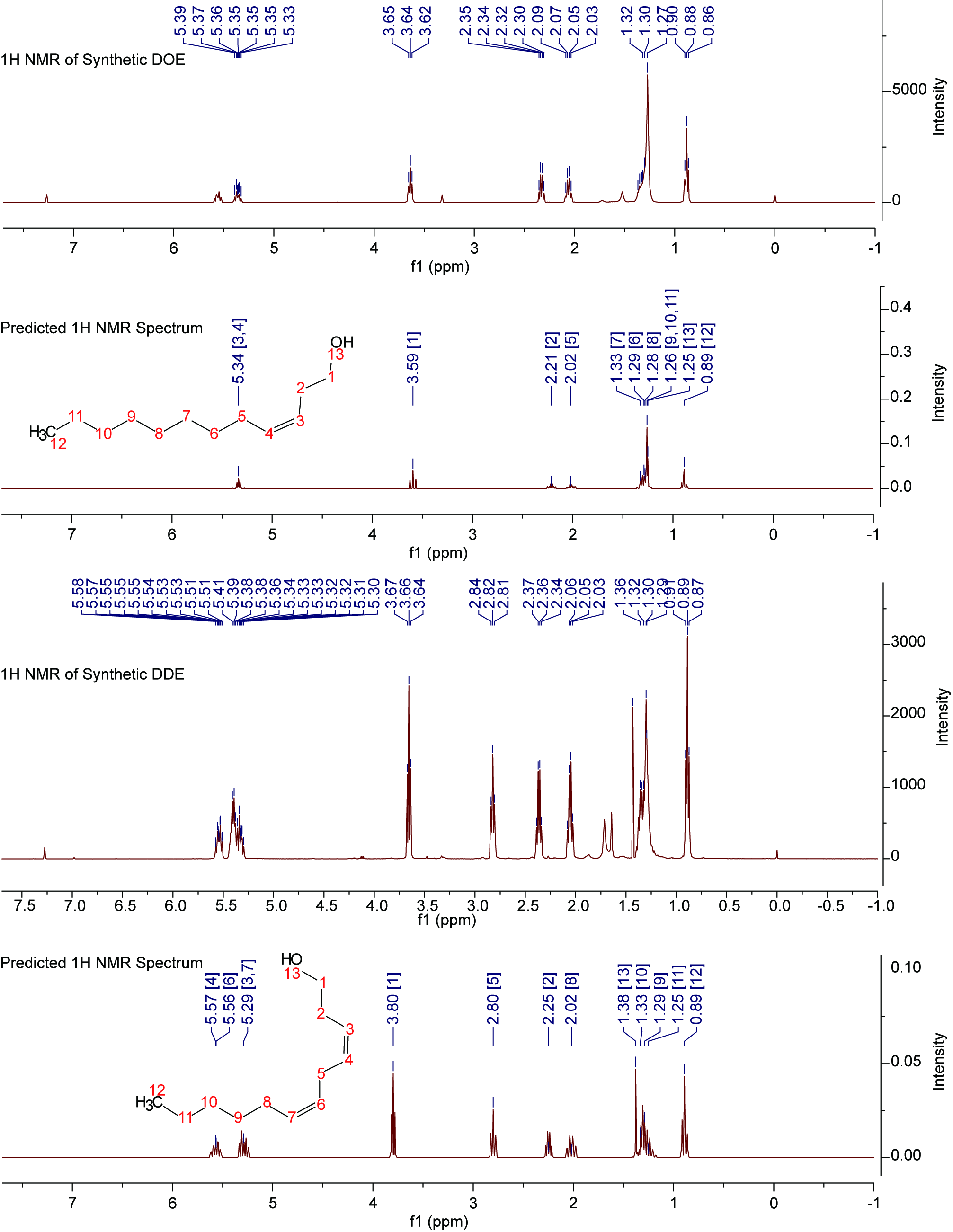

Supplement: Figure S4 — 1HNMR spectra of synthetic standards. (TIF) [file pone.0090906.s004.tif]

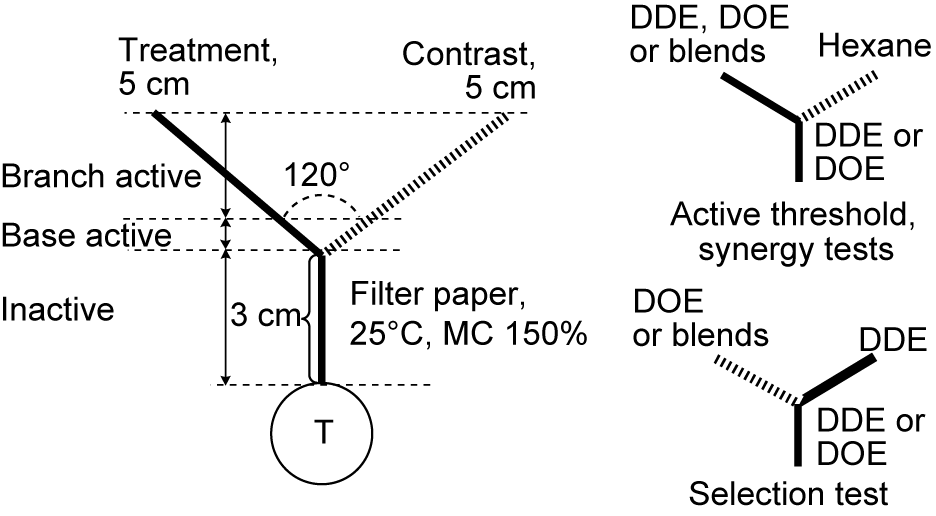

Supplement: Figure S5 — Trail-following bioassays. A Y-shape trail-following bioassay apparatus comprising a piece of filter paper and a glass waiting chamber where a termite (T) was deposited. Trails were drawn with microsyringes containing sternal gland extract, (3Z)-dodec-3-en-1-ol (DOE), (3Z,6Z)-dodeca-3,6-dien-1-ol (DDE), or hexane control according to the testing purpose (active threshold, synergy test or selection test). (TIF) [file pone.0090906.s005.tif]
