# Supplementary material for: Trail Communication Regulated by Two Trail Pheromone Components in the Fungus-Growing Termite Odontotermes formosanus (Shiraki)
Source: PLoS One. 2014 Mar 26;9(3):e90906. doi: 10.1371/journal.pone.0090906 (PMC3966735; doi:10.1371/journal.pone.0090906)
Supplement: Table S1 — LRI calculation of the two sternal glandular specific components in O. formosanus . Since both components were polarized, retention times or LRIs would change a lot (SE<10/0.5 year) with the bleeding of stationary phase on a DB-WAX capillary column. (DOC) [file pone.0090906.s006.doc]

Table S1. LRI calculation of the two sternal glandular specific components in *O. formosanus*.

| Columns | Components | Retention Times (min) | | | | LRIs |
| --- | --- | --- | --- | --- | --- | --- |
| HP-5 |  | C14 | C15 |  | A/B |  |
|  | A | 20.87 | 23.28 | - | 22.05 | 1448.70 |
|  | B | 20.87 | 23.28 | - | 22.24 | 1456.73 |
|  |  |  |  |  |  |  |
| DB-WAX |  | C19 | C20 | C21 | A/B |  |
|  | A | - | 26.29 | 28.14 | 27.15 | 2046.17 |
|  |  | - | 38.42 | 41.49 | 39.74 | 2043.00 |
|  |  | - | 12.22 | 12.86 | 12.55 | 2051.02 |
|  |  |  |  |  |  |  |
|  | B | 24.34 | 26.29 | - | 26.19 | 1995.06 |
|  |  | 35.21 | 38.42 | - | 38.21 | 1993.46 |
|  |  | 11.56 | 12.22 | - | 12.22 | 1999.40 |

Retention time or LRI would change a lot (SE<10/0.5 year) with the bleeding of stationary phase on a DB-WAX capillary column.
