# Supplementary material for: Trail Communication Regulated by Two Trail Pheromone Components in the Fungus-Growing Termite Odontotermes formosanus (Shiraki)
Source: PLoS One. 2014 Mar 26;9(3):e90906. doi: 10.1371/journal.pone.0090906 (PMC3966735; doi:10.1371/journal.pone.0090906)
Supplement: Table S2 — Volatile contaminants identified in the GC-MS analysis. (DOC) [file pone.0090906.s007.doc]

Table S2. Volatile contaminants identified in the GC-MS analysis

| Retention Times (min) | Structure | Retention Times(min) | Structure |
| --- | --- | --- | --- |
| 7.55 | Nonanal | 10.49 | Methoxy-phenyl-oxime |
| 8.11 | Acetic acid | 10.81 | Hexamethyl-cyclotrisiloxane, |
| 9.29 | Hexadecane | 11.34 | 1-Ethylidene-1H-indene |
| 9.60 | Butanoic acid | 11.56 | Nonadecane |
| 9.67 | Dimethyl-silanediol | 13.41 | Heneicosane |
| 9.94 | Isovaleric acid | 14.05 | Dibenzofuran |
| 10.08 | Heptadecane |  |  |
